# The effects of maternal depression and maternal selective serotonin reuptake inhibitor exposure on offspring

**DOI:** 10.3389/fncel.2013.00073

**Published:** 2013-05-21

**Authors:** J. D. A. Olivier, H. Åkerud, H. Kaihola, J. L. Pawluski, A. Skalkidou, U. Högberg, I. Sundström-Poromaa

**Affiliations:** ^1^Department of Women's and Children's Health, Uppsala UniversityUppsala, Sweden; ^2^Center for Gender Medicine, Karolinska InstitutetStockholm, Sweden; ^3^GIGA-Neurosciences, University of LiègeLiège, Belgium

**Keywords:** 5-HTT, maternal depression, neurodevelopment, serotonin, SSRI

## Abstract

It has been estimated that 20% of pregnant women suffer from depression and it is well-documented that maternal depression can have long-lasting effects on the child. Currently, common treatment for maternal depression has been the selective serotonin reuptake inhibitor medications (SSRIs) which are used by 2–3% of pregnant women in the Nordic countries and by up to 10% of pregnant women in the United States. Antidepressants cross the placenta and are transferred to the fetus, thus, the question arises as to whether children of women taking antidepressants are at risk for altered neurodevelopmental outcomes and, if so, whether the risks are due to SSRI medication exposure or to the underlying maternal depression. This review considers the effects of maternal depression and SSRI exposure on offspring development in both clinical and preclinical populations. As it is impossible in humans to study the effects of SSRIs without taking into account the possible underlying effects of maternal depression (healthy pregnant women do not take SSRIs), animal models are of great value. For example, rodents can be used to determine the effects of maternal depression and/or perinatal SSRI exposure on offspring outcomes. Unraveling the joint (or separate) effects of maternal depression and SSRI exposure will provide more insights into the risks or benefits of SSRI exposure during gestation and will help women make informed decisions about using SSRIs during pregnancy.

The number of women using selective serotonin reuptake inhibitors (SSRIs) during pregnancy is increasing, although knowledge on long-term neurodevelopmental effects to the child is lacking. This review summarizes clinical and preclinical findings of how SSRI exposure during pregnancy affects child outcomes. Many clinical findings parallel aspects of the preclinical data, such as decreased gestational length, birth weight, pain responses, and social behavior, increased spontaneous abortion/mortality rate, risk of cardiac anomalies, anxiety, depression, and rapid eye movement (REM) sleep, and affected 5-HT metabolism, motor development, and hypothalamic-pituitary-adrenal (HPA) stress reactivity. However, antenatal depression also has been associated with long-term neurodevelopmental outcomes. This review therefore starts by describing effects on the offspring exposed to antenatal depression and will then focus on outcomes of SSRI exposure during pregnancy.

## Maternal depression

Women are at an increased risk of becoming depressed during pregnancy and in the postpartum period, especially when they have pre-existing psychiatric illnesses. In fact, depressive symptoms may occur more frequently during pregnancy than in the postpartum period (Suri et al., 2007). During pregnancy, ~20% of women report symptoms of depression (Patkar et al., 2004), and 4–7% of pregnant women suffer from major depressive disorder (Andersson et al., 2003; Gorman et al., 2004; Melville et al., 2010). Among women who experience postpartum depression, nearly 40% develop their symptoms during pregnancy (Johnson, 1997). Biological and psychosocial factors, such as the genetic setup of the mother, hormonal/reproductive history, current stressors, and life experiences, are known to be risk factors for development of antenatal depression (Miller and LaRusso, 2011). Antenatal depression has been associated with higher rates of poor pregnancy outcomes (such as pre-eclampsia and premature delivery), impaired fetoplacental function, decreased fetal growth, and neonatal complications (Orr and Miller, 1995; Kurki et al., 2000; Bonari et al., 2004; Jablensky et al., 2005; Wisner et al., 2009; El Marroun et al., 2012). However, while premature delivery and decreased fetal growth are established outcomes of antenatal depression (Henrichs et al., 2010), the influence is most profound in low-income countries and countries with great health inequalities (Grote et al., 2010). Antenatal depression is also associated with poor nutrition, obesity, smoking, alcohol, and drug abuse which all can have negative effects on the developing child (Andersson et al., 2004; Bonari et al., 2004).

Several neurodevelopmental outcomes have been reported in children exposed to antenatal or postpartum depression. While it has long been known that postpartum depression is associated with poor maternal-child attachment with long-term repercussions (Murray and Cooper, 1997), fewer studies have addressed the effects of antenatal depression. DiPietro et al. (2006) reported that antenatal depression improved the mental and motor development in 2-year-old children, indicating that moderate amounts of maternal adversity may optimize early child development. However, most other studies have found negative associations between antenatal depression and neurodevelopmental outcomes in children. For instance, antenatal depression has been associated with developmental delays in 18-month-old children (Deave et al., 2008), increased behavioral and emotional problems in 4-year-old children (O'Connor et al., 2002), increased anxiety in 6- to 9-year-old children (Davis and Sandman, 2012), and attention problems in children aged 3 and 4 (Van Batenburg-Eddes et al., 2012). Later on, also gender-related offspring effects have been reported. Hay et al. (2008) tested the effects of antenatal and postpartum depression on children's outcomes during adolescence and found that 42% of the antenatally depression-exposed and 46% of the postpartum depression-exposed adolescents displayed emotional disorders. Interestingly, the association between antenatal depression and emotional disorders was only significant in adolescent girls. Parenthetically this gender-related offspring differences hold true for postpartum depression as well. Following exposure to maternal postpartum depression increased internalizing and externalizing problems in 12-year-old children have been reported (Agnafors et al., 2012), where girls expressed more internalizing problems, and boys expressed more externalizing problems. Hay et al. (2008) conclude that the greater the extent of exposure to maternal depression, the more likely it was for the child to develop a broader range of problems.

It should also be emphasized that paternal depression is of relevance for offspring developmental outcomes. Paulson et al. (2009) studied language development in children whose mother or father were depressed 9 and 24 months after birth. Depressive symptoms in either the mother or the father lowered the frequency of reading to their child. However, only fathers' depression predicted lower frequencies of reading to the child at the age of 24 months and reduced expressive language at the age of 2 years. Furthermore, van den Berg et al. (2009) showed that paternal depression also has an influence on excessive infant crying.

Thus, antenatal maternal depression poses a threat to maternal both well-being and healthy development in the offspring. These effects are likely due to a number of factors such as the physiology of the intrauterine environment, perinatal maternal and paternal mood disorders, current stressors, social support, timing, intensity, and genetic background. Therefore, understanding the influence of antenatal depression during pregnancy on child outcomes is rather complex. Incorporating methods of studying the fetus that has been exposed to antenatal depression provides the opportunity to examine the intrauterine milieu as the developmental niche of the fetus and will help us to unravel the mechanisms underlying maternal psychological factors that may have long-lasting developmental effects (DiPietro, 2012; Sandman and Davis, 2012).

## Antidepressant medication use during pregnancy

Continuing or starting pharmacological therapy during pregnancy is often unavoidable. Cohen et al. (2006) showed that 68% of depressed women who discontinued treatment relapsed during pregnancy, while only 26% of those who continued treatment did so. Currently, 1–3% of pregnant women in Europe are using antidepressant medications (El Marroun et al., 2012; ADs; Kieler, 2012), while user frequencies in the U.S. are 4–13% (Cooper et al., 2007; Hayes et al., 2012). Twenty-five percent of women on antidepressants continue treatment during pregnancy and 0.5% of pregnant women who have not been treated with antidepressants previously begin treatment (Ververs et al., 2006). As antidepressant medications cross the placenta and are evident in breast milk, questions have been raised about their developmental safety (Heikkinen et al., 2003; Noorlander et al., 2008). However, exposure to antenatal depression similarly increases the risk of child psychopathology (affective, anxiety, and disruptive behavior disorder; Weissman et al., 2006). Therefore, the question arises as to whether children exposed to maternal antidepressants are at risk and, if so, whether the risks are due to medication or to the underlying depression.

## Selective serotonin reuptake inhibitors

**Figure 1:**
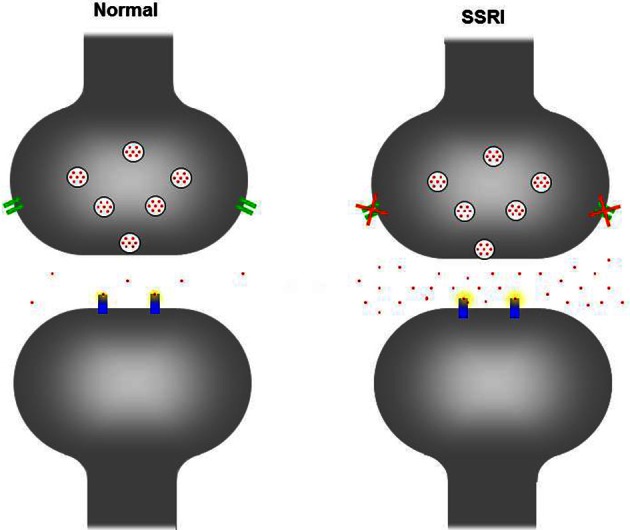
**Schematic figure of the serotonergic neuron in a normal situation (left) and when exposed to SSRIs (right).** Upon neuronal activation 5-HT (red dots) is being released in the extracellular cleft, activating receptors (blue) at the postsynaptic neuron. To end the signal, excessive 5-HT is being re-uptaken by the serotonin transporter (green) from the extracellular cleft into the presynaptic neuron. In the presynaptic neuron 5-HT is degraded and/or stored in vesicles for future release. In the picture on the right, the serotonin transporter has been blocked by a SSRI and is no longer capable to reuptake 5-HT in the presynaptic neuron, increasing the 5-HT in the extracellular cleft.

SSRIs are the most widely prescribed antidepressants worldwide because of their efficacy, relatively few (adverse) side-effects, and therapeutic safety (Barbey and Roose, 1998). SSRIs do not cause gross structural neuroteratogenic effects and are often considered to be safe for antenatal use (Gentile, 2005). Therefore, prescription of SSRIs during pregnancy, to promote the psychological health of the mother, has increased (Oberlander et al., 2006). By blocking the serotonin transporter (5-HTT) SSRIs inhibit the reuptake of serotonin (5-HT) into presynaptic nerve terminals resulting in an increase in the synaptic concentration of 5-HT (see Figure 1). During adulthood 5-HT mainly acts as a modulatory neurotransmitter regulating emotion, stress responses, arousal, sleep, learning, cognition, and attention. However, during brain development 5-HT also acts as a neurotrophic factor, regulating cell division, differentiation, migration, growth cone elongation, myelination, synaptogenesis, and dendritic pruning (Gaspar et al., 2003). Thus, changes in the 5-HT levels during neurodevelopment have the potential to affect a number of processes (Ansorge et al., 2007). While human studies are hampered by time and ethical constraints, animal models offer the possibility to study both the short- and long-term consequences of maternal SSRI exposure. Therefore, both **clinical** and **preclinical** data on the effects of maternal SSRI exposure on the offspring are described in this review.

### Clinical findings

Antidepressants are able to cross the placenta and relevant concentrations have been detected in umbilical vein blood (Hendrick et al., 2003b). Fluoxetine and citalopram have a high ratio of umbilical vein-to-maternal serum concentration, while sertraline and paroxetine have a low ratio. Maternal plasma levels of fluoxetine and its metabolite, norfluoxetine, decrease drastically during pregnancy (Heikkinen et al., 2003), probably due to the normal physiological changes during pregnancy. At birth, neonatal plasma levels of fluoxetine and norfluoxetine have been shown to be 65 and 72% of the maternal levels (Heikkinen et al., 2003).

#### Pregnancy complications

Two meta-analyses have revealed that SSRIs and other antidepressant medications may increase the risk of miscarriage (Hemels et al., 2005; Rahimi et al., 2006). However, this may not always be the case (reviewed in Ellfolk and Malm, 2010), while a recent meta-analysis showed only a borderline association (Ross et al., 2013). Women continuing SSRI use after the first trimester also have an increased risk of preeclampsia compared with women who discontinue treatment or non-users (Qiu et al., 2009; Toh et al., 2009b; Reis and Källén, 2010). Recently, Palmsten et al. (2012) found that the risk of developing preeclampsia was similar in non-depressed and depressed women (2.3 and 2.4%, respectively). Furthermore, compared to depressed women, the relative risk of preeclampsia after SSRI exposure in GW10 and 20 was 3.3 for monotherapy and 4.5 for polytherapy [and even greater for selective noradrenalin reuptake inhibitors (SNRI) and tricyclic antidepressants (TCA)]. In conclusion, antidepressant use during pregnancy increases the risk of preeclampsia, with modest effects after use of SSRIs and much higher effects after use of SNRIs and TCAs.

#### Pregnancy outcomes

As with maternal antenatal depression, SSRI use during pregnancy has often been associated with increased rate of preterm birth (Chambers et al., 1996; Costei et al., 2002; Simon et al., 2002; Källén, 2004; Wen et al., 2006; Davis et al., 2007; Lund et al., 2009; Wisner et al., 2009; Reis and Källén, 2010; Yonkers et al., 2012), decreased birth weight (Chambers et al., 1996; Källén, 2004; Wen et al., 2006), being born small for gestational age (Oberlander et al., 2006; Toh et al., 2009a), and reduced fetal head growth (El Marroun et al., 2012). However, several studies did not find an effect of SSRIs on preterm birth (Kulin et al., 1998; Ericson et al., 1999; Suri et al., 2004; Malm et al., 2005; Oberlander et al., 2006; Toh et al., 2009a) and birth weight (Ericson et al., 1999; Suri et al., 2004; Malm et al., 2005; Lund et al., 2009; Reis and Källén, 2010). Nevertheless, an inverse relationship was found between lower gestational age and high doses of SSRIs in late pregnancy (Suri et al., 2007). Several theories have been postulated for low birth weight after exposure to SSRIs; for example, fluoxetine reduces maternal appetite and weight gain, which may affect fetal growth (Chambers et al., 1996). However, other SSRIs have been associated with weight gain, rather than weight loss. Another theory is that the altered 5-HT levels, caused by SSRIs use, increase the risk of intrauterine growth retardation and preterm delivery by impairing placental blood flow (Wen et al., 2006). Whether or not these factors play a role in gestational age and weight remains to be elucidated.

#### Umbilical cord blood monoamine and metabolite concentrations

SSRI treatment during pregnancy reduces whole blood 5-HT (−69%), 5-hydroxyindoleacetic acid (−18%; 5-HIAA; main metabolite of 5-HT) and homovanillic acid (−23%; a major catecholamine metabolite) concentrations in the umbilical vein (Laine et al., 2003). In infants, lower 5-HIAA concentrations are inversely correlated with 5-HTergic symptom scores (such as myoclonus, restlessness, tremor, shivering, hyperreflexia, incoordination, and rigidity) and there is a positive correlation between cerebrospinal fluid and peripheral blood 5-HT/metabolite concentrations (Sarrias et al., 1990). This suggests an association between the central 5-HTergic effects and the cord blood 5-HIAA concentration. Similarly, plasma levels of noradrenalin were decreased in the umbilical vein of SSRI-exposed infants and there was also a tendency for reduced dihydroxyphenylglycine (DHPG; group I metabotropic glutamate receptor selective agonist) and 3,4-Dihydroxyphenylacetic acid (DOPAC; metabolite of dopamine) in SSRI-exposure infants (Laine et al., 2003). Not surprisingly, pharmacokinetic differences exist between antidepressants. DHPG concentrations were significantly lower (−40%) in fluoxetine-exposed infants compared with citalopram-exposed infants. This effect may be due to the lower affinity of citalopram, compared to fluoxetine, for the noradrenaline reuptake pump (Hyttel, 1994). On the other hand, citalopram, but not fluoxetine, significantly reduces cord blood DOPAC concentrations compared with controls. Thus, maternal use of SSRIs induces significant changes in the cord blood 5-HT and metabolite concentrations. However, it remains to be determined how these changes in 5-HT and its metabolite impact the outcome of the offspring.

#### Neonatal adaptation

In the first 2 weeks after birth up to 30% of antenatal SSRI-exposed neonates display poor neonatal adaption such as respiratory distress, temperature instability, feeding difficulties, jitteriness, irritability, sleep problems, tremors, shivering, restlessness, convulsions, rigidity, hypoglycaemia, and jaundice (Chambers et al., 1996; Cohen et al., 2000; Costei et al., 2002; Casper et al., 2003; Laine et al., 2003; Källén, 2004; Oberlander et al., 2006; Davis et al., 2007). These effects occur more often in neonates who were exposed to SSRIs in late pregnancy, and symptoms arise earlier and more often in neonates exposed to higher SSRI doses (Costei et al., 2002; Källén, 2004; Davis et al., 2007). A dose-response effect of paroxetine on neonatal adaptation problems has been reported (Levinson-Castiel et al., 2006) with higher doses of paroxetine being related to greater neonatal adaptation problems. In addition to the dose, the duration of SSRI exposure plays a significant role on neonatal outcomes with respiratory distress being linked to longer prenatal SSRI exposure (Oberlander et al., 2008c). It is unclear whether the neonatal adaptation symptoms are a result of neonatal withdrawal from the SSRIs or overstimulation of the 5-HTergic system (Isbister et al., 2001). Nevertheless, these symptoms are usually mild and disappear 2 weeks postpartum (Moses-Kolko et al., 2005).

Another way to measure neonatal adaptation is to measure gross outcome markers such as Neonatal Intensive Care Unit (NICU) admission and neonatal seizures. Several studies report an increased risk of neonatal seizures, longer hospital stays, and NICU admissions after SSRI use during pregnancy (Simon et al., 2002; Källén, 2004; Lattimore et al., 2005; Oberlander et al., 2006; Wen et al., 2006; Cole et al., 2007; Davis et al., 2007), although an increased risk for NICU admissions have also been found after prenatal depression (Chung et al., 2001). Malm et al. (2005) found that 11.2% of neonates exposed to SSRIs in the first trimester and 15.7% of infants exposed to SSRIs during the third trimester of pregnancy were treated in specialized or intensive care units. There is also a 2- to 8-fold increase risk for low Apgar scores in SSRI-exposed neonates (Källén, 2004; Lund et al., 2009; Wisner et al., 2009). Neonates of depressed mothers also often display low Apgar scores (Wisner et al., 2009). Therefore, it is difficult to disentangle whether the low Apgar scores and NICU admissions are due to the SSRI exposure or to the underlying depression.

#### Congenital malformations in the neonate

SSRI use during pregnancy may increase the risk for congenital malformations and cardiac anomalies. A Danish study reported that 4.9% of infants exposed to SSRIs during the first trimester of pregnancy, and 6.8% exposed to SSRIs during late pregnancy display congenital malformations, while corresponding the figure in non-exposed infants was 3.4% (Wogelius et al., 2006). Chambers et al. (1996) found more minor anomalies in infants exposed to SSRIs during the first trimester of pregnancy compared with non-exposed infants, while no differences were found in the number of major anomalies. Alwan et al. (2007) report that first trimester SSRI exposure increase**s** the risk for anencephaly, craniosynostosis, and omphalocele. Louik et al. (2007) also found an increased risk for omphalocele and for septal defects after first trimester exposure to sertraline and an association between paroxetine exposure and right ventricular outflow tract obstruction defects. Moreover, sertraline was associated with anal atresia and limb-reduction defects and paroxetine was associated with neural tube defects, club foot, and undescended testes (Louik et al., 2007). Cardiac malformations were also reported by Malm et al. (2005) and Diav-Citrin et al. (2008), who found a 3- to 4-fold increased in cardiac malformations in infants of fluoxetine-exposed women. However, there are also several studies that do not report an association with maternal prenatal SSRI exposure and neonatal congenital malformations (Altshuler et al., 1996; Ericson et al., 1999; Simon et al., 2002; Hendrick et al., 2003a; Einarson and Einarson, 2005; Källén and Otterblad, 2007). Overall, the effects of prenatal SSRI exposure on congenital malfunction appear small and seem to be most apparent when SSRIs are used in the first trimester of pregnancy. However, the effects of prenatal SSRIs on congenital heart disease becomes more severe if SSRIs are taken with other medications, such as benzodiazepines (Oberlander et al., 2008d).

#### Persistent pulmonary hypertension in the neonate

In the condition of persistent pulmonary hypertension (PPHM) the pulmonary vasculature fails to relax after birth, which results in hypoxemia. The occurrence of PPHN is ~0.2% in live-born infants and it is associated with substantial infant mortality and morbidity. Several studies have shown an increased risk for PPHM in SSRI-exposed infants. Exposure during the first trimester (Källén and Olausson, 2008), as well as during late pregnancy (Chambers et al., 1996, 2006), significantly increases the risk for PPHM. This result was confirmed in a large Nordic study, where the risk for PPHM in neonates after SSRI exposure was shown to be at least doubled (Kieler, 2012). However, several studies did not find any association between prenatal SSRI use and PPHM (Andrade et al., 2009; Wichman et al., 2009; Wilson et al., 2011). Moreover, both maternal depression and SSRI usage have been linked to increased risk of premature birth (Wisner et al., 2009), with the risk of PPHN being four times higher in babies born at 34–36 weeks compared to those with full-term gestation (Källén and Otterblad, 2007; Hibbard et al., 2010). Therefore, it is difficult to state whether maternal SSRI exposure truly increases the risk for PPHM, or if other, secondary, factors contribute to the increased risk for PPHM.

#### Neurodevelopmental outcomes

Within the first week after birth, infants are exposed to a routine heel lance (blood sampling for screening of metabolic diseases). Oberlander et al. (2002) used this acute noxious event to study the effect of maternal SSRI exposure on neonatal responses to pain. In response to the heel lance, SSRI-exposed newborns show significantly less facial activity and a reduced heart rate, indicating that prenatal exposure to SSRIs attenuates the response to acute pain in newborns. When the heel lance was repeated after 2 months, the pain response was still attenuated in SSRI exposed infants (Oberlander et al., 2005). The attenuated pain response may be due to increased 5-HT and GABA agonist levels caused by SSRIs, as 5-HT and GABA agonists are known to play a role in pain modulation and are active during early fetal neurologic growth (Whitaker-Azmitia, 2001; Oberlander et al., 2002). Zeskind and Stephens (2004) found that SSRI-exposed infants displayed increased tremulousness, fewer changes in behavioral state, fewer different behavioral states and greater amounts of uninterrupted REM-sleep. Together, these results suggest that prenatal SSRI exposure has an effect which already appears early after birth.

Although some studies exist, the long-term neurodevelopmental outcomes of prenatal SSRI exposure have not been extensively studied. With regards to language development, Nulman et al. (1997); Nulman et al. (2002) studied the IQ, temperament and language development in children (16 and 86 months old) who were exposed to SSRIs during pregnancy but did not find any effects of prenatal SSRI exposure on the neurodevelopmental outcomes measured. Prenatal SSRI exposure also appears to have no effect on motor or speech development during the first 2 years of life (Simon et al., 2002). Interestingly, Weikum et al. (2012) compared infants of healthy mothers, with infants exposed to SSRIs and infants exposed to antenatal depression and found that SSRI-exposed infants showed accelerated perceptual development by discriminating both vowels and consonants at 36 weeks gestation (while *in utero*). These data indicate that SSRI-exposure may alter the developmental time course of language perception.

However, there are several studies which describe an effect of prenatal SSRI exposure on neurobehavioral outcomes. Oberlander et al. (2007) studied externalizing behaviors (attention, aggression, attention/hyperactivity, and oppositional or defiant behaviors) in 4 year olds and found that SSRI-exposed children had greater externalizing scores than the clinical cutoff. Data on internalizing behaviors is more conflicting. Whereas prenatal SSRI exposure and/or maternal depression have been associated with increased internalizing behaviors (e.g., depression, anxiety, withdrawal) in 3- and 4-year-old children (Oberlander et al., 2010), other studies have found no such effects (Misri et al., 2006). Additional studies report that 6–40 month old SSRI-exposed children show mild effects on motor development and control (tremulousness and fine motor movements), and lower Psychomotor Developmental Index (PDI) scores on the Bayley Scales of infant development (Casper et al., 2003). Mortensen et al. (2003) studied psychomotor development in 7- to 10-month-old children by means of the Boels test and found that in children prenatally exposed to antidepressants (not specific for SSRIs) had an increased risk for abnormal Boels test, indicating that the risk for abnormal psychomotor development (such as hearing, sight, and motor attention) is higher in children exposed to antidepressants. Recently prenatal SSRI exposure, especially during the first trimester, has been associated with an increased risk for autism spectrum disorders (Croen et al., 2011). Together these data suggest that prenatal SSRI exposure has effects on neurodevelopmental outcomes, at birth and also later in childhood.

#### Stress regulation

Apart from its role in neurodevelopment, 5-HT is implicated in the development and function of the HPA axis (Meaney et al., 1994; Laplante et al., 2002; Andrews and Matthews, 2004) and prenatal SSRI exposure has been suggested to affect aspects of HPA function. Previous work has shown that prenatal SSRI exposure results in attenuated basal salivary cortisol levels (Brennan et al., 2008; Oberlander et al., 2008b) and attenuated facial action and heart rate in response to an acute painful stressor in infants (Oberlander et al., 2002, 2005). Corticosteroid binding globulin (CBG), a transporter and regulator of circulating cortisol levels (Siiteri et al., 1982), has been shown to be increased in SSRI-exposed neonates, particularly after vaginal delivery (Pawluski et al., 2012a). This increase in neonatal CBG levels was negatively associated with diurnal changes in salivary cortisol at 3 months of age. Furthermore, infants prenatally exposed to SSRIs have lower evening basal cortisol levels and there are lower post-stress cortisol levels in non-SSRI exposed and non-breastfed infants compared with SSRI-exposed and non-SSRI exposed infants who were breastfed at 3 months of age (Oberlander et al., 2008b). These findings suggest that the effect of prenatal SSRI exposure is present, but may only become apparent in a particular maternal caregiving context (Hanley and Oberlander, 2012).

#### Serotonin transporter gene

The 5-HT transporter (5-HTT) plays a critical role in moderating environmental influences and developmental risks (Homberg and Lesch, 2011). Humans carry a polymorphism in the promoter region of the 5-HTT gene (5-HTTLPR), which involves a common 44-base pair insertion/deletion of a repetitive sequence (Lesch et al., 1996). The dominant short (S) allelic variant reduces transcriptional efficiency of the SERT as compared with the long (L) allelic variant (Lesch et al., 1996). Allelic variation of 5-HTTLPR may contribute to the responsiveness of SSRIs in depressed patients. Pollock et al. (2000) showed that paroxetine reduced depressive symptoms more rapidly in patients with the LL genotype compared with S-allele carriers. Even early in life allelic variation of the 5-HTTLPR can influence neonatal behavior, especially in combination with environmental factors. For example, when maternal anxiety levels were high, more negative emotionality was found in infants carrying the S-allele, whereas no effect of the 5-HTTLPR was found in circumstances with low maternal anxiety (Pluess et al., 2011). Tiemeier et al. (2012) also showed that the effect of maternal anxiety during fetal life and early adulthood is moderated by the 5-HTTLPR of the child. Children with the S-allele were at increased risk of developing emotional problems and were less accurate in emotion-matching, indicating affected ability to process emotions. Adults with two S-alleles may be at increased risk for depression following early life adversity (Caspi et al., 2003; Kendler et al., 2005; Lesch, 2007); however, under positive environments S-allele carriers might benefit more compared to L-allele carriers. Hankin et al. (2011) showed that positive parenting resulted in higher levels of positive affect in S-allele infants. These data are in agreement with the theory of Belsky et al. (2009) who suggested that S-allele carriers are more vulnerable in general, not only negatively, but also positively. Thus, vulnerability genes, or risk alleles, seem to make individuals more susceptible to environmental influences.

The combination of the allelic variation in 5-HTTLPR and prenatal SSRI exposure may compound risks associated with altered 5-HT levels. Recently an association was found, after prenatal SSRI exposure, between (1) SS-allele carriers and lower 5-min Apgar score and risk for neuromotor symptoms; (2) LS-allele carriers and low birth weight; and (3) LL-allele carriers and respiratory distress and tachypnea (Oberlander et al., 2008a). In 3-year old SS-allele carriers, prenatal exposure to maternal anxiety was associated with increased internalizing behaviors and in 3-year old LL carriers, prenatal maternal anxiety was associated with more externalizing behaviors, regardless of prenatal SSRI exposure (Oberlander et al., 2010). Thus, 5-HTTLPR genotype influences the effect of antenatal mood on child behavior (Oberlander et al., 2010) and may modulate the outcome of adverse neonatal effects following maternal SSRI exposure. However, much more research is necessary to understand how perinatal exposure to SSRIs affect developmental outcomes and how these effects differ from the effects of exposure to perinatal maternal mood disorders.

### Preclinical findings

In order to better understand the neurobehavioral and long-term effects of perinatal exposure to SSRIs animal models have been used. In particular, much research has investigated these effects using rodents. At birth rats and mice are at a relative early stage of maturation and their brain maturation occurs after birth. This makes rodents highly suitable as a model for studying the direct effects of SSRI exposure on early brain development. When rats and mice are between 12 and 13 days old, the maturation of the cerebral cortex is comparable to the human neocortex around birth (Romijn et al., 1991; Homberg et al., 2010). The first and second trimester of pregnancy in humans is comparable to the prenatal period in rats, while the third trimester in humans is comparable to the period right after birth (until PND12–13) in rats. In the following studies both prenatal exposure and postnatal exposure to SSRIs are described.

#### Pregnancy outcomes

SSRIs are able to cross the placenta in rodents at a similar transfer rate to humans. Noorlander et al. (2008) exposed mice (i.p. injection) from embryonic day (E)8–E16 of gestation with either fluoxetine or fluvoxamine and collected blood plasma 5 h after the last injection. The transfer rate of fluoxetine across the placenta in mice (69%) was similar to the transfer rate of fluoxetine across the placenta in women (73%). A lower placental transfer rate was found for fluvoxamine in both mice (30%) and humans (35%). When pregnant rats were injected daily with fluoxetine from gestational day (G)11 until birth the placental transfer rate 5 h after the last injection was 83% for fluoxetine and 78% for norfluoxetine (Olivier et al., 2011). The norfluoxetine/fluoxetine ratio was 1.44 in mothers and 1.39 in pups, which is similar to the ratios found in humans (Lundmark et al., 2001). SSRIs are able to pass the blood brain barrier (Baumann and Rochat, 1995) and this was confirmed in the study of Olivier et al. (2011). Both fluoxetine and norfluoxetine have been detected in whole brain samples of rat pups (Olivier et al., 2011). Although differences exist between transfer rates of different SSRIs, they are transferred from mother to pup, altering both the periphery and the central nervous systems. At the highest dose of fluoxetine tested (0.8 mg/kg/day), an 81% mortality rate was found after prenatal exposure, while fluvoxamine did not affect the survival rate in mice (Noorlander et al., 2008). A 10-fold higher mortality rate of neonatal rats was also found after prenatal paroxetine exposure (van den Hove et al., 2008). Interestingly, rats that were prenatally exposed to fluoxetine (12 mg/kg/day; orally) from E11 until birth did not show increased mortality (Olivier et al., 2011). However, litters that were prenatally exposed to fluoxetine were smaller, therefore prenatal mortality is possible. Prenatal paroxetine exposure in rats did not influence the litter size at birth, but did reduce the gestational length and birth weight (van den Hove et al., 2008). Prenatal fluoxetine exposure from E11 until birth did not affect the gestational length, but did reduce the weight of pups early after birth (Olivier et al., 2011). Interestingly, Vorhees et al. (1994) have found increased neonatal mortality after prenatal fluoxetine exposure. Days of exposure and the use of different rat strains may account for differences between studies. No effects were found on long-term growth or survival (Vorhees et al., 1994; Olivier et al., 2011). In conclusion, differences types of SSRIs, doses, time-periods of SSRI exposure, and animal strains likely influence the birth and neonatal outcomes.

#### Monoamine and biochemical concentrations

Prenatal exposure to fluoxetine from E11 to E21 significantly reduced placental levels of 5-HT in rats (Fornaro et al., 2007). Postnatal exposure to Zimelidine (SSRI) to rat pups 2–3 weeks after birth significantly increased the 5-HIAA/5-HT ratio in the brain stem and cortex of 2 month old offspring (Hilakivi et al., 1995). In prenatally stressed mice, treatment with fluoxetine during postnatal weeks 1–3 also lowered the 5-HT turnover rate in offspring (Ishiwata et al., 2005). These data indicate that the 5-HT metabolism is affected by early SSRI exposure both in the periphery and the central nervous system. Limited amounts of information are available on the biochemical profile in rodents prenatally exposed to SSRIs. The neonatal behavioral syndrome, which is often seen after withdrawal of SSRIs, is associated with hypoglycemia (Favreliere et al., 2010). For this reason Dubovický et al. (2012) studied glucose, lactate dehydrogenase, aspartate aminotransferase/alanine aminotransferase ratio and antioxidant status in blood from prenatally (E15–E20) venlafaxine-exposed (SNRI) rats. However they report no differences between venlafaxine-exposed and non-exposed rat offspring on postnatal day (PND)21.

#### Congenital malformations

A higher mortality rate has been found in neonatal rodents after prenatal SSRI exposure (Noorlander et al., 2008; van den Hove et al., 2008) and it has been postulated that heart malformations may be one reason for this increase in mortality. Noorlander et al. (2008) found that the majority of fluoxetine-exposed offspring died postnatally because of severe dilated cardiomyopathy. Moreover, the ratio of thickness of the left ventricle to the radius of the left ventricle cavity was significantly decreased in prenatal fluoxetine-exposed mouse offspring both at PND20 and during adulthood. These data clearly show that prenatal fluoxetine exposure (0.8 mg/kg/day; i.p.) severely affects heart development, resulting in an increased death rate in offspring. *In vitro*, (Sari and Zhou, 2003) found that paroxetine significantly decreased the rate of proliferation of fetal heart cells (E13) from rats, particularly cardiac myocytes and, to a lesser degree, non-muscle cells. Fluoxetine and sertraline also have similar influences on the proliferation of cardiac cells in the mouse embryo (Yavarone et al., 1993). These data indicate that changes in prenatal 5-HT levels influence the proliferation of the embryonic heart cells, at least *in vitro*. Fluoxetine has furthermore been shown to affect cell viability and differentiation from undifferentiated ES cells to cardiomyocytes in a dose-dependent manner. Analysis of tissue-specific markers showed also that fluoxetine inhibits mesodermal development but it promotes ectodermal differentiation (Kusakawa et al., 2008). In another study, late two-cell stage embryos incubated with fluoxetine for 6 h were more likely to develop into blastocysts compared to the controls. Exposure to fluoxetine for 24 h showed a reduction in blastocyst formation, suggesting a time dependent effect of fluoxetine on blastocyst formation. It also appears that these effects are, in part, due to altered TREK signaling (Kim et al., 2012). In humans, the cardiomyocyte proliferation is essentially complete at birth, whereas in rodents cardiomyocyte growth and proliferation is robust for the first 14 days after birth (Clubb and Bishop, 1984; Walsh et al., 2010). Haskell et al. (2012) injected mouse offspring with sertraline from PND1 to PND14, reflecting the third trimester in humans, and found that sertraline-exposed offspring showed increased heart rate and activity levels, as well as smaller left ventricular internal diameters in diastole and decreased stroke volumes, indicating changes in the cardiac morphology. Taken together, both *in vitro* and *in vivo* early-exposure to SSRIs have adverse consequences for the developmental outcomes of the heart.

#### Pulmonary hypertension

As far as we know, only one study has investigated the effects of prenatal SSRI exposure on pulmonary hypertension in animal models (Fornaro et al., 2007). Fluoxetine exposure during late gestation resulted in abnormal oxygenation and a higher mortality rate in new-born rat pups compared to non-exposed controls. Moreover, the right ventricular mass of the lung was higher in prenatal fluoxetine-exposed rats compared to controls. Interestingly the effects seem to be sex-dependent; the right ventricular hypertrophy after prenatal fluoxetine exposure was only significant in female pups (Belik, 2008). Moreover, the thickness of the medial smooth muscle layer of the small and large pulmonary arteries (used as magnitude of pulmonary vascular modeling) tended to be thicker in the female, compared to male, pups. These sex-differences in rats are interesting as the prevalence for PPHN in humans is higher in male infants (Hernandez-Diaz et al., 2007).

Rodents that constitutively lack the 5-HTT could be seen as a model for life-long SSRI exposure from conception. In 5-HTT knockout (5-HTT^−/−^) mice that were exposed to hypoxia for several weeks, the number and wall thickness of pulmonary vessels decreased compared with controls (Eddahibi et al., 2000). Moreover, compared with wild-type controls the right ventricular systolic pressure was lower and the right ventricle hypertrophy was less hypertrophied in hypoxic 5-HTT^−/−^ mice. In mice that overexpress the 5-HTT (5-HTT+) there is a 3-fold increase in right ventricle pressure compared to wild-type mice (MacLean et al., 2004). Moreover, when 5-HTT+ mice were exposed to hypoxia, right ventricular hypertrophy and pulmonary vascular remodeling were doubled compared to wild-types (MacLean et al., 2004).

In summary, SSRI exposure during development increases the risk for pulmonary hypertension in rodent models. Moreover, overexpression of the 5-HTT from conception on increases the risk, while disruption of the gene lowers the risk, for pulmonary hypertension. It appears that the imbalance of the 5-HTT during development contributes to the development of pulmonary hypertension.

#### Neurodevelopmental outcomes

Prenatal fluoxetine exposure (G6–G20) has been reported to cause a transient delay in motor development in rats on PND10 and PND12; decreased horizontal activity in an open arena on PND8, but increased retention time on a rotating rod on PND22 and PND49 (Bairy et al., 2007). With respect to pain, the sensitivity in response to a hot-plate test on PND30, PND45, and PND70 was not altered by early fluoxetine exposure (G0-PND21) in mice (Lisboa et al., 2007) or after fluoxetine exposure (PND1–21) in 8-week-old male rat offspring (Knaepen et al., 2013). However, in adolescent rat offspring postnatal fluoxetine exposure (PND0–PND6) did reduce pain sensitivity (Lee, 2009). Moreover, sensorimotor learning deficits were found in adolescence offspring exposed to fluoxetine, as well as reduced dendritic complexity of thalamocortical afferents and in layer IV of the barrel cortex on PND7 (Lee, 2009). In line with this, Xu et al. (2004) showed that early postnatal paroxetine exposure (PND0–PND8) in rats disrupts the organization of thalamocortical somatosensory barrels on PND8. Recent work has also shown that adult male offspring exposed postnatally (PND1–21) to fluoxetine has increased post-operative pain, measured as hypersensitivity to mechanical stimuli after hind paw incision (Knaepen et al., 2013). However, fluoxetine exposure to prenatally stressed offspring normalized post-operative pain. This suggests that the actions of fluoxetine likely differ in the presence of maternal adversity (Knaepen et al., 2013). Taken together, these data suggest that early SSRI exposure alters cortical development resulting in impaired transmission of tactile information to the primary somatosensory cortex.

Sleep-wakefulness patterns are also altered by early SSRI exposure. Escitalopram exposure (PND5–PND19) increased REM-sleep duration and decreased REM latency in mouse offspring (Popa et al., 2008). In rat offspring, postnatal chlorimipramine exposure (week 1–3) resulted into reduced active sleep, compensated with quiet sleep (Mirmiran et al., 1981). Apart from altered sleep patterns, chlorimipramine-exposed animals also performed less efficiently on a temporal learning task but responded more rapidly in a spatial alternation learning task. Prenatal exposure to fluoxetine (G6–G20) increased cognitive performance; fluoxetine-exposed rat offspring found a hidden platform in a water maze faster compared with controls and had an increased latency to enter a compartment where they previously received a shock (Bairy et al., 2007). Using a model of prenatal stress, Ishiwata et al. (2005) found that postnatal fluoxetine treatment (postnatal weeks 1–3) to mouse offspring reduced the deficits in spatial learning and memory seen after prenatal stress. Moreover, postnatal SSRI exposure reversed the prenatal stress-induced reduction in spine and synapse density in CA3 pyramidal cells of the hippocampus (Ishiwata et al., 2005). As the learning ability strongly correlates with the spine or synapse density in hippocampal neurons, these data indicate that the increased synapse density found after early fluoxetine exposure is the cellular basis of restoring learning deficits induced by prenatal stress. Together these data indicate a favorable effect of early SSRI exposure on learning and memory.

With respect to social and reproductive behaviors, early (G0 to PND21) fluoxetine exposure (Lisboa et al., 2007) as well as postnatal (PND1–PND19) citalopram exposure (Manhães de Castro et al., 2001) increased the latency to the first attack of an intruder, indicating reduced aggression. Postnatal treatment (PND8–PND21) with chlorimipramine, a tricyclic antidepressant, clearly disturbed the performance of sexual behavior in male offspring with fewer mice ejaculating (Mirmiran et al., 1981). The offspring that did ejaculate showed an increased latency to the first ejaculation. Nevertheless, the number of mounts and intromissions were similar between groups, although the mount/intromission ratio was higher in chlorimipramine-exposed animals indicating that these animals were less efficient. Maciag et al. (2006) found that postnatal citalopram exposure (PND8–PND21) significantly impaired mounting behavior, reduced the number of intromissions and the number of ejaculations. Interestingly, when rats were prenatally (G11 till birth) exposed to fluoxetine no effects were found on the sexual performance (Olivier et al., 2011). However, developmental fluoxetine treatment (PND1–21) decreased the anogenital distance in juvenile male offspring, decreased the number of intromissions, increased the latency to the first intromission, and increased the latency to the first ejaculation in sexually naive male offspring (Rayen et al., 2013). These effects were not evident if postnatal fluoxetine exposure occurred after prenatal stress. Furthermore, developmental fluoxetine and/or prenatal stress decreased the area of the sexually dimorphic nucleus of the preoptic area (SDN-POA) in these offspring (Rayen et al., 2013). Prenatal fluoxetine exposure significantly affected juvenile play behavior and, during adulthood, prenatal fluoxetine-exposed animals still tended to make less contact with other rats (Olivier et al., 2011). Postnatal exposure (PND8–PND21) to citalopram also decreased the interest to play in male, but not female, juvenile rats (Simpson et al., 2011). In conclusion, social and reproductive behaviors appear to be most affected when 5-HT levels are disturbed during the postnatal period in rodent models.

Affective behaviors in offspring are also altered by early SSRI exposure. When rats were postnatally (PND8–PND21) exposed to citalopram a neophobic response to an auditory stimulus, as well as reduced exploration to a novel object, were found (Simpson et al., 2011). In addition, citalopram exposure led to abnormal myelin formation and a reduction in callosal connectivity, indicating the importance of normal 5-HT homeostasis for a proper maturation of the brain. Both prenatal (Bairy et al., 2007; Olivier et al., 2011) and postnatal (Mirmiran et al., 1981; Ansorge et al., 2004; Lisboa et al., 2007; Ansorge et al., 2008; Popa et al., 2008; Simpson et al., 2011) SSRI exposure increased anxiety-like behaviors in adult mice and rats. Also depression-like behavior was increased after prenatal (Olivier et al., 2011) and postnatal (Hansen et al., 1997; Lisboa et al., 2007; Popa et al., 2008) SSRI exposure in adulthood. In adolescence, recent work has shown that postnatal fluoxetine exposure (PND1–21) does not significantly alter depressive-like behavior in male and female rat offspring (Rayen et al., 2011). In addition, postnatal fluoxetine exposure reversed effects of prenatal stress on depressive-like behavior in adolescent offspring, thus normalizing this behavior (Rayen et al., 2011). Similarly, postnatal fluoxetine exposure reversed the effects of prenatal stress on hippocampal neurogenesis in adolescence (Rayen et al., 2011). This suggests that the long-term effects of fluoxetine may vary with age and previous exposure to maternal stress.

The 5-HT_1A_ receptor might be an important factor contributing to the altered affective behaviors. During early brain development, the 5-HT_1A_ receptor is involved in neurite branching (Sikich et al., 1990), neurite outgrowth and neuronal survival (Fricker et al., 2005). Moreover, 5-HT_1A_ autoreceptors in raphe 5-HTergic neurons are important in regulating central 5-HT neurotransmission by their negative feedback of 5-HT neuron firing. Functional desensitization of the 5-HT_1A_ autoreceptors is one of the mechanisms that **is** thought to play a role in the therapeutic action of SSRIs (Pineyro and Blier, 1999). Interestingly, both prenatal (Olivier et al., 2011) and postnatal (Popa et al., 2008) SSRI exposure increased the 5-HT_1A_ agonist-induced hypothermia, indicating increased sensitivity of the 5-HT_1A_ receptor. Besides changes in the 5-HT_1A_ receptor functioning, embryonic SSRI exposure has also been shown to reduce 5-HTT expression (Hansen and Mikkelsen, 1998) and 5-HT_2_ receptor density and function (Cabrera and Battaglia, 1994). Thus, early exposure to SSRIs affects the 5-HTergic system, however, processes downstream of 5-HT receptors also mediate the neurotrophic effect of 5-HT. Moreover epigenetic modifications may contribute to developmental outcomes (Kinnally et al., 2010). Overall, early exposure to SSRIs has an effect on brain development and neuroplasticity (for review see: Pawluski, 2012) which can markedly alter the behavior of the offspring.

#### Stress regulation

Prenatal SSRI exposure has been shown to affect the developing HPA system in animal models. For example, prenatal exposure to fluoxetine increased cortisol levels in fetal lambs (Morrison et al., 2004). Moreover, postnatal exposure to SSRIs decreased the serum corticosterone levels and reduced the expression of CA3 hippocampal glucocorticoid receptor (GR) and its co-activator GR interacting protein 1 (GRIP1) in adolescent rat offspring (Pawluski et al., 2012b). These results were only found in male adolescent offspring, indicating a sex difference in the neurodevelopmental outcome. Postnatal exposure to fluoxetine (weeks 1–3) was also shown to reverse the effects of prenatal stress on the corticosterone response to stress in adult mouse offspring (Ishiwata et al., 2005). Postnatal fluoxetine exposure to prenatally stressed rats also increased CBG levels during adolescence, suggesting significant alterations in circulating levels of free corticosterone (Pawluski et al., 2012b). Of interest is the fact that these results were sex specific with long-term effects of combined early-life stress and fluoxetine exposure on the HPA system existing only in male offspring. These sex differences are likely due to differences in circulating sex steroid hormone levels, as estradiol has been shown to modulate the HPA system (Viau and Meaney, 1991; Atkinson and Waddell, 1997; Viau, 2002). Much more research is necessary to unravel the mechanisms underlying these sex differences in HPA development and the role of steroid hormones and monoamines in regulating these effects.

#### Serotonin transporter gene

The polymorphism in the promoter of the 5-HTT is unique for primates and not present in rodents (Caspi et al., 2010), but the role of the 5-HTT has been extensively studied in rodent models with genetic deletion of the 5-HTT (Murphy and Lesch, 2008; Kalueff et al., 2010; Homberg and Lesch, 2011). The phenotypes observed in these 5-HTT knockout (5-HTT^−/−^) rodents mimic the long-term behavioral outcomes of early SSRI exposure. 5-HTT^−/−^ rodents display reduced pain, exploratory behavior, social behavior, and increased anxiety-like and depression-like behavior (Kalueff et al., 2010). Moreover, 5-HTT^−/−^ rodents have improved cognitive performance (Brigman et al., 2010; Nonkes et al., 2011; Van den Hove et al., 2011; Nonkes et al., 2012). Regarding neuronal plasticity, SERT^−/−^ rodents have reduced brain-derived neurotrophic factor and activity-regulated cytoskeleton associated protein expression levels in hippocampus and prefrontal cortex (Molteni et al., 2009, 2010). Moreover, neuronal PAS domain protein 4, regulating activity-dependent genes and neuroprotection, is reduced in SERT^−/−^ rodents and this effect could be mimicked by prenatal fluoxetine exposure (Guidotti et al., 2012). Reduced densities and functional alterations of 5-HT receptors have been found in SERT^−/−^ rats, as well as changes in neurodevelopment (reviewed in: Kalueff et al., 2010). The overlapping findings of life-long 5-HTT ablation and early-life exposure to SSRIs in rodents suggest that neurodevelopmental changes are responsible for the phenotypes observed. Therefore, the 5-HTT^−/−^ model is of heuristic value in studying the neurodevelopmental outcome of SSRI exposure.

## Concluding remarks

This review summarized clinical and preclinical findings of how SSRI exposure during pregnancy affects child outcomes. Although many clinical findings parallel aspects of the preclinical data (Table 1), in preclinical studies SSRIs are often administered to healthy animals, while in the clinic SSRIs are only administered to depressed women. Moreover, preclinical models are often tested during adulthood, whereas most clinical data comes from children. These factors should be taken into account.

**Table 1:** **Overview of clincal and preclinical findings after early SSRI exposure**.

	**Humans**	**Prenatal exposure in rodents**	**Postnatal exposure in rodents**
Pregnancy complications	↑ spontaneous abortion^1^^,^^2^	↑ mortality rate in newborn rats^7^	↑ mortality rate in mice^8^
	↑ risk of preeclampsia^3^^−^^6^		↑ mortality rate in rats^9^
Pregnancy outcomes	↑ rate of preterm birth^5^^,^^10^^−^^17^and ↓ gestational length^18^	↓ litter size and ↓ weight of the pups in rats^22^	↓ gestational length and birth weight in rats^9^
	↓ birth weight^10^^,^^13^^,^^16^, ↑ small for gestational age^19^^,^^20^, and ↓ fetal head growth^21^		
Monoamines and metabolites	↓ concentrations of 5-HT, 5-HIAA, and HVA in whole blood^23^	↓ 5-HT levels in the placenta of rats^7^	affected 5-HT metabolism in the periphery and central nervous system^24^^,^^25^
	↓ concentration of noradrenalin, DHPG and DOPAC in infants^23^		
Congenital malfunctions	↑ risk for congenital malformations like anencephaly, craniosynostosis, omphalocele, and septal defects^10^^,^^26^^−^^28^	↓ proliferation rate of embryonic heart cells in rat and mice^32^^,^^33^	severe dilated cardiomyopathy and ↓ ratio of the left ventricle thickness/the left ventricle cavity radius in mice^8^
	↑ risk of cardiac anomalies^27^^,^^29^^−^^31^		↑ heart rate and activity levels in mice,
			↓ left ventricular internal diameters in diastole and ↓ stroke volumes in mice^34^
Persistent pulmonary hypertension (PPHM)	↑ risk of PPHM in infants^10^^,^^35^^−^^37^	abnormal oxygenation^7^	↓ number and wall thickness of pulmonary vessels and ↓ right ventricle hypertrophy in hypoxic 5-HTT^−/−^ mice^39^
		↑ right ventricular mass of the lung and thicker medial smooth muscle layer of the small and larger pulmonary arteries of female rats^38^	↑ in right ventricular hypertrophy and pulmonary vascular remodeling in hypoxic 5-HTT+ mice^40^
Neurodeve-lopmental outcomes	↓ response to acute pain in newborns^41^	influence motor development transiently in rats^47^	↓ pain response^57^
	↑ tremulousness, ↓ changes in behavioral state and ↓ different behavioral states in infants^42^	↑ cognitive performance and ↓ impulsivity (treatment from G0 until PND21)^47^^,^^48^	↑ REM sleep duration, ↓ REM latency and ↓ active sleep in rodents^52^^,^^53^
	↑ amounts of uninterrupted REM sleep in infants^42^	↓ juvenile play behavior^22^	↓ efficient performance on a temporal learning task, but more rapidly in the spatial alternation learning task^52^
	mild effects on motor development and motor control^43^	↓ contact making with other rats and ↑ self-grooming behavior^22^	recovered learning deficits in MWM and reversed altered hippocampal spine and synapse density in prenatally stressed mice^25^
	affected psychomotor development in younger children^44^	↑ anxiety-like behavior^22^^,^^47^	↑ sensorimotor learning deficits^57^
	↑ internalizing behaviors in early adulthood^45^	↑ depression-like behavior^22^	↓ juvenile play behavior in male rats^54^
	↑ risk for autism spectrum disorders^46^	↑ 5-HT1A agonist-induced hypothermia^22^	↓ aggressive behavior in rats^55^
		↓ 5-HTT expression^49^ and 5-HT2 receptor density and function^50^	↓ sexual activity and performance^52^^,^^56^
			↑ anxiety-like behavior^48^^,^^52^^−^^54^^,^^57^^,^^58^
			↑ depression-like behavior^48^^,^^53^^,^^59^
			↑ the 5-HT_1A_ receptor agonist-induced hypothermia^53^
			↓ dendritic complexity of thalamocortical afferents and layer IV of the barrel cortex^51^
			disrupts the organization of thalamocortical somatosensory barrels^60^
Stress regulation	↓ basal salivary cortisol levels, diurnal changes in salivary cortisol and altered HPA stress reactivity patterns^61^^,^^62^		↓ serum corticosterone levels and ↓ expression of CA3 hippocampal GR and GRIP1 in healthy male rats^65^
	↓ increased heart rate in response to acute painful stressors^41^^,^^63^		↑ CBG levels and normalized corticosterone response to stress in postnatal SSRI-treated prenatally stressed rodents^25^^,^^65^
	↑ CBG levels in serum of neonates^64^		

References:

1 Hemels et al., 2005;

2 Rahimi et al., 2006;

3 Palmsten et al., 2012;

4 Qiu et al., 2009;

5 Reis and Källén, 2010;

6 Toh et al., 2009a,b;

7 Fornaro et al., 2007;

8 Noorlander et al., 2008;

9 van den Hove et al., 2008;

10 Chambers et al., 1996;

11 Costei et al., 2002;

12 Davis et al., 2007;

13 Källén, 2004;

14 Lund et al., 2009;

15 Simon et al., 2002;

16 Wen et al., 2006;

17 Wisner et al., 2009;

18 Suri et al., 2007;

19 Oberlander et al., 2006;

20 Toh et al., 2009a,b;

21 El Marroun et al., 2012;

22 Olivier et al., 2011;

23 Laine et al., 2003;

24 Hilakivi et al., 1995;

25 Ishiwata et al., 2005;

26 Alwan et al., 2007;

27 Louik et al., 2007;

28 Wogelius et al., 2006;

29 Diav-Citrin et al., 2008;

30 Malm et al., 2005;

31 Oberlander et al., 2008a,b,c,d;

32 Sari and Zhou, 2003;

33 Yavarone et al., 1993;

34 Haskell et al., 2012;

35 Chambers et al., 2006;

36 Kieler, 2012;

37 Källén and Olausson, 2008;

38 Belik, 2008;

39 Eddahibi et al., 2000;

40 MacLean et al., 2004;

41 Oberlander et al., 2002;

42 Zeskind and Stephens, 2004;

43 Casper et al., 2003;

44 Mortensen et al., 2003;

45 Oberlander et al., 2010;

46 Croen et al., 2011;

47 Bairy et al., 2007;

48 Lisboa et al., 2007;

49 Hansen and Mikkelsen, 1998;

50 Cabrera and Battaglia, 1994;

51 Lee, 2009;

52 Mirmiran et al., 1981;

53 Popa et al., 2008;

54 Simpson et al., 2011;

55 Manhães de Castro et al., 2001;

56 Maciag et al., 2006;

57 Ansorge et al., 2004;

58 Ansorge et al., 2008;

59 Hansen et al., 1997;

60 Xu et al., 2004;

61 Brennan et al., 2008;

62 Oberlander et al., 2008a,b,c,d;

63 Oberlander et al., 2005;

64 Pawluski et al., 2012a;

65 Pawluski et al., 2012b.

In addition there are often discrepancies between clinical findings and this may be due the trimester when SSRIs are taken, whether other medications were also administered, variety of other diagnoses (e.g., anxiety), the dose of the medication and the gestational age of the infant. In preclinical studies, discrepancies between findings may be due to the timing of SSRI exposure (prenatal or postnatal), the duration of exposure, the dose administered and the SSRI used, as well as rodent strain.

Both genetic and environmental factors contribute to the well-being of a child. In humans, it is impossible to study the effects of SSRI exposure without taking the underlying depression into account. In animals, it is possible to disentangle the effects of maternal depression from the effects of maternal SSRI exposure. Moreover, the timing of maternal adversity and SSRI exposure (duration and dosing) can be studied during the prenatal or postnatal period or during both periods. The additional advantages of using animal models are that one can readily examine long-term neurodevelopmental outcomes, specific roles of maternal care, and neural plasticity. Unfortunately, most preclinical research to date has studied the effects of SSRIs in healthy animals. In order to make preclinical findings translational, it is important to study the effects of SSRIs in a model of maternal depression or adversity, as the actions of developmental exposure to SSRIs can significantly vary with exposure to maternal adversity. Finally, preclinical studies reveal sexually dimorphic responses which likely apply to humans as well. It is, therefore, important to take the sex of the offspring into account.

It remains to be determined whether maternal SSRI use is more beneficial or has adverse effects beyond the underlying depression. Much more research is needed to understand the risks and benefits of perinatal exposure to SSRIs on the developing child. Future research should focus on the effects of maternal depression alone, and compare it to offspring exposed to SSRIs, and offspring exposed to SSRIs combined with maternal adversity. Unraveling the different underlying mechanisms (which can be environmental, genetic, or epigenetic) in these three different groups will provide the answer for the risks and benefits of SSRI use during pregnancy.

### Conflict of interest statement

The authors declare that the research was conducted in the absence of any commercial or financial relationships that could be construed as a potential conflict of interest.
